# Public understanding of medical cannabis in Poland 7 years after legalization: findings from a cross-sectional study

**DOI:** 10.3389/fpubh.2025.1686709

**Published:** 2025-09-22

**Authors:** Andrzej Silczuk, Mateusz Jankowski, Paulina Mularczyk-Tomczewska, Agata Olearczyk, Tomasz Baran, Iwona Wrześniewska-Wal, Aleksandra Lewandowska, Magdalena Łoś

**Affiliations:** ^1^Department of Community Psychiatry, Faculty of Health Sciences, Medical University of Warsaw, Warsaw, Poland; ^2^Department of Population Health School of Public Health Centre of Postgraduate Medical Education, Warsaw, Poland; ^3^Department of Public Health, Faculty of Health Sciences, Medical University of Warsaw, Warsaw, Poland; ^4^Health Innovation Unit, SGH Warsaw School of Economics, Warsaw, Poland; ^5^Department of Business Psychology and Social Innovation, University of Warsaw, Warsaw, Poland; ^6^School of Public Health Centre of Postgraduate Medical Education, Warsaw, Poland; ^7^J. Babinski Specialist Psychiatric Health Care Team, Psychiatric Ward of Children Lodz, Łódź, Poland; ^8^Department of Social Medicine and Public Health, Medical University of Warsaw, Warsaw, Poland

**Keywords:** medical cannabis, legalization, therapeutic awareness, cannabinoid therapy, sociodemographic factors, patient education

## Abstract

**Background:**

Medical cannabis has been legally available in Poland since 2017, yet its integration into routine clinical practice remains limited. This study investigates public attitudes toward medical cannabis, therapeutic awareness, and the perceived readiness of the healthcare system 7 years after legalization.

**Methods:**

A cross-sectional survey was conducted in July 2025 using the computer-assisted web interviewing (CAWI) method on a nationally representative sample of 1,113 adults (aged 18–84). The questionnaire assessed opinions on the legalization of medical cannabis, willingness to undergo cannabinoid-based therapy, perceptions of physician and patient knowledge, and support for home cultivation under medical supervision. Multivariable logistic regression was used to identify sociodemographic predictors of key attitudes.

**Results:**

A substantial majority of respondents supported the legalization of medical cannabis (81.1%) and expressed willingness to undergo treatment if medically indicated (84.3%). However, only 4.2% reported having received a recommendation for medical cannabis from a physician. Confidence in physicians’ (29.9%) and patients’ (16.1%) knowledge about medical cannabis was low. Oncological conditions (57.4%) and chronic pain (49.8%) were the most frequently recognized therapeutic indications. Support for home cultivation was associated with prior medical cannabis use, male gender, younger age, and urban residence. Older adults (≥50 years) were more likely to support legalization, whereas those aged 30–39 and individuals with moderate household income were less accepting.

**Conclusion:**

While public support for medical cannabis in Poland is high, its clinical implementation remains limited. Bridging this gap will require comprehensive educational initiatives and evidence-based guidelines to support healthcare professionals and inform patients.

## 1 Introduction

Medical cannabis has undergone a significant resurgence in global healthcare policy and practice. Despite its historical classification as a controlled substance under the 1961 United Nations Single Convention on Narcotic Drugs, which restricted its medical use across Europe, North America, the United Kingdom, and Australia, many countries have since implemented legislation legalizing cannabis based medicines and medical cannabis for therapeutic purposes (1) and this shift reflects growing interest in the potential clinical benefits of cannabinoids.

This policy transformation has not only expanded therapeutic access but has also influenced population-level patterns of cannabis use worldwide (2). A 2024 meta-analysis encompassing data from 33 European, 15 American, and 16 Asian countries demonstrated that jurisdictions with legalized medical or recreational cannabis exhibit markedly higher lifetime prevalence of cannabis use, 12.0% (95% CI: 10.0–14.3), compared to 5.4% (95% CI: 4.3–6.9) in non-legalized regions (3). While systematic reviews suggest that cannabis based medicines and medical cannabis may provide modest relief for conditions such as anxiety, nausea, insomnia, and osteoarthritis related pain, the overall quality of supporting evidence remains heterogeneous, often limiting the strength of clinical recommendations.

In Poland, medical cannabis was legalized in November 2017 (with implementation in 2018), positioning the country among the earlier European adopters (2). Seven years post legalization, a mixed methods study found that most patients perceive prescription access as relatively straightforward. Nonetheless, persistent concerns regarding treatment costs, product quality, and chemovar consistency remain prevalent (4).

At the same time, data collected from clinicians highlight a different perspective: significant barriers to prescribing persist, largely related to systemic and educational gaps. Surveys indicate that over 60% of Polish physicians have received no formal training in cannabinoid based therapies, and more than 70% report feeling inadequately prepared to counsel patients on cannabis based medicines use (2). Despite approximately 54% of physicians encountering patient inquiries about medical cannabis in the preceding 6 months, only around 8% have ever issued a prescription (5). To date, no clinical trials conducted in Poland have definitively confirmed the efficacy and safety of medical cannabis for specific therapeutic indications; consequently, treatment decisions rely primarily on international evidence and the individual clinical judgment of prescribing physicians (6). These findings underscore an urgent need for evidence based clinical guidelines and comprehensive educational initiatives to support safe and effective integration of medical cannabis into the routine medical care. The lack of strong, evidence-based clinical guidelines mainly reflects the limited number of high-quality randomized trials and real-world evidence studies on cannabis and cannabinoid therapies. Overcoming regulatory, commercial, and structural barriers to such research is crucial to build the evidence needed for guideline development, especially given the growing interest in their therapeutic potential across diverse indications (7).

Based on current data from European reviews and monitoring reports, the prevalence of medical cannabis use and the number of prescriptions issued vary significantly across European countries. These differences reflect diverse regulatory frameworks, product availability, and levels of clinical awareness. In Germany, which has had one of the most advanced publicly reimbursed medical cannabis systems since 2017, the number of patients using medical cannabis increased from approximately 1,000 prior to legalization to over 60,000 in 2019, resulting in more than 185,000 prescriptions issued annually (8). Similar trends have been observed in Italy, where national monitoring data (2013–2019) indicate an increase from approximately 400 to nearly 13,000 patients per year, with over 26,000 prescriptions issued in 2019 (8).

Comparative studies across European Union countries suggest that legality and availability do not automatically translate into widespread therapeutic use. Restrictive regulations, lack of reimbursement, and limited physician training continue to pose barriers to access. A review involving 17 European countries found that products such as nabiximols are relatively accessible, whereas the use of herbal cannabis remains hindered by legal and logistical obstacles (9).

Additional data from the Liguria region of Italy demonstrate a steady increase in medical cannabis utilization following the implementation of an integrated clinical and organizational model that improved coordination between physicians, pharmacists, and regional health authorities (10).

Exploring the divergence between clinical medical cannabis policies and legal frameworks across Europe is essential, as recent analysis shows that while some countries regulate cannabis strictly for medical purposes, they often maintain decriminalization rather than full legalization, highlighting a nuanced interplay that impacts patient access and regulatory oversight (11).

Since the legalization of medical cannabis in Poland in 2017, the topic has gained growing visibility in clinical, regulatory, and societal debates. However, despite the increasing number of prescriptions and expanding therapeutic indications, little is known about how the Polish public understands and evaluates the role of cannabis-based therapies. The primary aim of this study was to assess public support for the therapeutic use of medical cannabis, including attitudes toward its legalization and individual readiness to undergo cannabinoid treatment.

A secondary objective was to explore public perceptions of risks, clinical awareness, patient rights, and professional knowledge related to medical cannabis.

## 2 Materials and methods

### 2.1 Study design and sample characteristics

This study employed a cross-sectional design based on a structured survey distributed online to a representative sample of adult Polish residents. Data collection took place between 11th and 13th of July 2025, using the computer-assisted web interviewing (CAWI) technique. Respondents were recruited via the Ariadna online research panel, which comprises over 110,000 active members aged 18 years and older and is routinely calibrated to reflect the demographic structure of the Polish population.

To ensure national representativeness, a stratified sampling procedure was used. The initial stratification divided the population into exclusive subgroups based on demographic criteria such as sex, age, type of residence, geographical region, and level of education. Sampling quotas were then matched to population data derived from Statistics Poland (Główny Urząd Statystyczny, GUS), the official institution responsible for demographic and social statistics at the national and regional levels.

Participation in the survey was entirely voluntary, and all respondents provided informed consent prior to participation. The study team developed the conceptual framework and constructed the survey tool. Invitations were distributed individually by email, with a subsequent reminder sent via text message. The survey required completion of all items, ensuring a dataset without missing responses. The final response rate was approximately 22%. Data were collected anonymously and processed in accordance with ethical standards. The study protocol received ethical approval from the Bioethics Committee of the Medical University of Warsaw (approval no. AKBE/39/2025, dated: Febr. 24, 2025).

### 2.2 Questionnaire and measures

The study instrument was a structured questionnaire developed specifically for this research, drawing on recent literature concerning medical cannabis policy, therapeutic use, and public health communication. The analytical scope was limited to seven closed-ended questions that addressed key domains relevant to public perception, therapeutic awareness, and health system readiness in relation to medical cannabis.

These items explored respondents’ evaluations of the 2017 legalization of medical cannabis in Poland, their willingness to use cannabinoid-based treatments if recommended by a physician, and concerns about potential unintended consequences such as increased recreational use. Further questions assessed knowledge of medical indications, support for home cultivation under clinical supervision, and perceptions of informational adequacy among both physicians and patients.

All items utilized a five-point Likert scale format (ranging from “definitely not” to “definitely yes”) or multiple-choice response sets. The questionnaire was pilot tested on non-medical adults from the general population for clarity, internal logic, and cultural appropriateness. Recent studies have employed a similar methodological approach (12–14). Minor modifications were introduced following cognitive debriefing to optimize comprehensibility. The final instrument required approximately 8 min to complete.

Sociodemographic information collected from respondents included gender, age, educational attainment (primary, secondary, or higher), type and size of place of residence (rural area; town <20,000; town 20,000–99,999; city 100,000–499,999; city >500,000), and occupational status. These variables enabled stratified analysis of cannabis-related attitudes across population subgroups.

## 3 Results

Completed questionnaires were received from 1,113 adults aged 18–84 years, 54.4% were females. Detailed characteristics of the study population are presented in Table 1.

**Table 1:** Characteristics of the study population (*n* = 1,113).

Variable		n	%
Gender	Female	605	54.4
	Male	508	45.6
Age group [years]	18–29	153	13.7
	30–39	217	19.5
	40–49	211	19.0
	50–59	205	18.4
	60+	327	29.4
Educational level	primary	18	1.6
	Vocational	116	10.4
	Secondary	443	39.8
	Higher	536	48.2
Marital status	Single	319	28.7
	Married	580	52.1
	Informal relationship	167	15.0
	Other	47	4.2
Place of residence	Rural area	419	37.6
	City below 20,000 residents	145	13.0
	City from 20,000 to 99,999 residents	219	19.7
	City from 100,000 to 499,999 residents	187	16.8
	City ≥ 500,000 residents	143	12.8
Number of household members	1 (living alone)	191	17.2
	2	405	36.4
	3 or more	517	46.5
Occupational status	Active	695	62.4
	Passive	418	37.6
Self-reported household economic status	Good	525	47.2
	Moderate	416	37.4
	Bad	172	15.5

Among the respondents, 81.1% declared positive (35.5% rather positively and 45.6% definitely positive) opinion towards the legalization of medical cannabis treatment for patients in Poland since 2017 (Table 2). The majority of respondents (84.3%) reported that they would accept medical cannabis therapy if diagnosed with a condition for which such treatment is recommended. A total of 4.2% of respondents reported having ever received medical cannabis treatment. Less than half of respondents (45.5%) declared support the possibility of patients growing medical cannabis for personal therapeutic use with physician approval. Only 29.9% of respondents believed that physicians in Poland possess sufficient knowledge regarding medical cannabis therapy, or were unsure about the extent of physicians’ knowledge. Furthermore, just 16.1% believed that patients are adequately informed about the therapeutic potential of medical cannabis, or were uncertain in this regard. Oncological conditions (57.4%) and chronic pain (49.8%) were the most recognized medical indications (diseases or conditions) for medical cannabis use. Public attitudes towards the use of medical cannabis are presented in Table 2.

**Table 2:** Public attitudes towards the use of medical cannabis (*n* = 1,092).

Item	Variable	n	%
How do you perceive the legalization of medical cannabis treatment for patients in Poland since 2017?	Definitely negatively	13	1.2
	Rather negatively	35	3.1
	Rather positively	395	35.5
	Definitely positively	508	45.6
	Difficult to say	162	14.6
If you were diagnosed with a condition for which medical cannabis is a recommended treatment and your physician prescribed it, would you agree to such therapy?	Definitely no	18	1.6
	Rather no	36	3.2
	Rather yes	372	33.4
	Definitely yes	566	50.9
	Difficult to say	121	10.9
Has a physician ever recommended medical cannabis therapy to you?	Yes	47	4.2
	No	1,066	95.8
Do you support the possibility of patients growing medical cannabis for personal therapeutic use with physician approval?	Definitely no	165	14.8
	Rather no	210	18.9
	Rather yes	253	22.7
	Definitely yes	254	22.8
	Difficult to say	231	20.8
In your opinion, do physicians in Poland have sufficient knowledge about medical cannabis therapy?	Definitely no	105	9.4
	Rather no	282	25.3
	Rather yes	251	22.6
	Definitely yes	81	7.3
	Difficult to say	394	35.4
In your opinion, do patients in Poland have sufficient knowledge about the potential use of medical cannabis therapy?	Definitely no	213	19.1
	Rather no	444	39.9
	Rather yes	137	12.3
	Definitely yes	42	3.8
	Difficult to say	277	24.9
In your opinion, for which medical indications (diseases or conditions) is medical cannabis used? (Select all that apply)	Chronic pain	554	49.8
	Neurological disorders (e.g., epilepsy and others)	431	38.7
	Oncology (cancer-related pain, chemotherapy-induced nausea and vomiting, cachexia)	639	57.4
	Psychiatric disorders (e.g., PTSD, stress, anxiety, insomnia, depression)	299	26.9
	Inflammatory and autoimmune diseases (e.g., rheumatic diseases, inflammatory bowel diseases)	224	20.1
	Fatigue, lack of energy	101	9.1
	Difficulty concentrating	84	7.5
	ADHD	123	11.1
	Lack of motivation	80	7.2
	I am not aware of any medical indications for the use of medical cannabis	239	21.5

Multivariable logistic regression analyses (Tables 3–7) were performed to identify factors associated with public perception of medical cannabis therapy.

**Table 3:** Factors associated with public perception of the legalization of medical cannabis treatment for patients in Poland since 2017 (*n* = 1,113).

How do you perceive the legalization of medical cannabis treatment for patients in Poland since 2017?—“rather positively” or “definitely positively”				Bivariable logistic regression		Multivariable logistic regression	
Variable		**%**	*p*	OR (95%CI)	*p*	aOR (95%CI)	*p*
Gender	Female (*n* = 605)	79.7	0.2	0.81 (0.60–1.10)	0.2		
	Male (*n* = 508)	82.9		Reference			
Age group [years]	18–29 (*n* = 153)	75.2	**0.04**	Reference		Reference	
	30–39 (*n* = 217)	77.4		1.13 (0.70–1.84)	0.6	1.25 (0.76–2.04)	0.4
	40–49 (*n* = 211)	81.0		1.14 (0.85–2.34)	0.2	1.58 (0.95–2.64)	0.08
	50–59 (*n* = 205)	82.9		1.61 (0.96–2.69)	0.1	1.73 (1.02–2.92)	**0.04**
	60 + (*n* = 327)	85.3		1.92 (1.19–3.10)	**0.01**	2.19 (1.34–3.56)	**0.002**
Educational level	Higher (*n* = 536)	83.4	0.06	1.33 (0.98–1.81)	0.06		
	Less than higher (*n* = 577)	79.0		Reference			
Marital status	Single (*n* = 319)	79.6	**0.04**	Reference			
	Married (*n* = 580)	83.8		1.32 (0.93–1.88)	0.1		
	Informal relationship (*n* = 167)	77.8		0.90 (0.57–1.42)	0.6		
	Other (*n* = 47)	70.2		0.60 (0.31–1.19)	0.1		
Place of residence	Rural area (*n* = 419)	79.7	0.5	0.87 (0.54–1.42)	0.6		
	City below 20,000 residents (*n* = 145)	78.6		0.82 (0.46–1.46)	0.5		
	City from 20,000 to 99,999 residents (*n* = 219)	84.9		1.25 (0.71–2.20)	0.4		
	City from 100,000 to 499,999 residents (*n* = 187)	81.3		0.97 (0.55–1.69)	0.9		
	City ≥ 500,000 residents (*n* = 143)	81.8		Reference			
Number of household members	1 (living alone) (*n* = 191)	79.1	0.7	0.87 (0.58–1.32)	0.5		
	2 (*n* = 405)	82.0		1.05 (0.75–1.47)	0.8		
	3 or more (*n* = 517)	81.2		Reference			
Occupational status	Active (*n* = 695)	81.0	0.9	0.98 (0.72–1.34)	0.9		
	Passive (*n* = 418)	81.3		Reference			
Self-reported household economic status	Good (*n* = 554)	82.0	**0.001**	0.79 (0.50–1.25)	0.3	0.73 (0.46–1.16)	0.2
	Moderate (*n* = 396)	75.7		0.54 (0.39–0.76)	**<0.001**	0.51 (0.37–0.71)	**<0.001**
	Bad (*n* = 142)	85.1		Reference		Reference	
Ever treatment with medical cannabis	Yes (n = 47)	91.5	0.06	2.58 (0.91–7.25)	0.07		
	No (*n* = 1,066)	80.7		Reference			

OR – odds ratio; aOR – adjusted odds ratio. Bold values are statistically significant.

**Table 4:** Factors associated with public perception of the treatment with medical cannabis (*n* = 1,113).

If you were diagnosed with a condition for which medical cannabis is a recommended treatment and your physician prescribed it, would you agree to such therapy?—“rather yes” or “definitely yes”				Bivariable logistic regression		Multivariable logistic regression	
Variable		%	*p*	OR (95%CI)	*p*	aOR (95%CI)	*p*
Gender	Female (*n* = 605)	82.5	0.07	0.74 (0.53–1.03)	0.07		
	Male (*n* = 508)	86.4		Reference			
Age group [years]	18–29 (*n* = 153)	85.0	**<0.001**	0.70 (0.40–1.23)	0.2	0.64 (0.35–1.17)	0.2
	30–39 (*n* = 217)	73.3		0.34 (0.21–0.54)	**<0.001**	0.33 (0.20–0.53)	**<0.001**
	40–49 (*n* = 211)	83.9		0.64 (0.39–1.07)	0.09	0.64 (0.38–1.06)	0.09
	50–59 (*n* = 205)	88.3		0.93 (0.54–1.62)	0.8	0.88 (0.50–1.53)	0.6
	60 + (n = 327)	89.0		Reference		Reference	
Educational level	Higher (*n* = 536)	84.5	0.8	1.04 (0.75–1.43)	0.8		
	Less than higher (*n* = 577)	84.1		Reference			
Marital status	Single (*n* = 319)	81.2	0.2	Reference		Reference	
	Married (*n* = 580)	86.4		1.47 (1.02–2.12)	**0.04**	1.23 (0.84–1.82)	0.3
	Informal relationship (*n* = 167)	83.2		1.15 (0.70–1.88)	0.6	1.15 (0.69–1.91)	0.6
	Other (*n* = 47)	83.0		1.13 (0.50–2.54)	0.8	0.95 (0.41–2.19)	0.9
Place of residence	Rural area (*n* = 419)	82.3	0.4	0.99 (0.60–1.63)	0.9		
	City below 20,000 residents (*n* = 145)	83.4		1.07 (0.58–1.98)	0.8		
	City from 20,000 to 99,999 residents (*n* = 219)	87.2		1.45 (0.80–2.60)	0.2		
	City from 100,000 to 499,999 residents (*n* = 187)	87.2		1.44 (0.78–2.64)	0.2		
	City ≥ 500,000 residents (*n* = 143)	82.5		Reference			
Number of household members	1 (living alone) (*n* = 191)	81.7	0.2	0.89 (0.58–1.37)	0.6		
	2 (*n* = 405)	86.7		1.30 (0.90–1.88)	0.2		
	3 or more (*n* = 517)	83.4		Reference			
Occupational status	Active (*n* = 695)	83.9	0.6	0.92 (0.66–1.29)	0.6		
	Passive (*n* = 418)	84.9		Reference			
Self-reported household economic status	Good (*n* = 554)	83.1	**0.001**	0.65 (0.40–1.05)	0.08	0.65 (0.39–1.07)	0.09
	Moderate (*n* = 396)	79.6		0.51 (0.36–0.73)	**<0.001**	0.51 (0.35–0.73)	**<0.001**
	Bad (*n* = 142)	88.4		Reference		Reference	
Ever treatment with medical cannabis	Yes (*n* = 47)	89.4	0.3	1.59 (0.62–4.09)	0.3		
	No (*n* = 1,066)	84.1		Reference			

OR – odds ratio; aOR – adjusted odds ratio. Bold values are statistically significant.

**Table 5:** Factors associated with public support for the possibility of patients growing medical cannabis for personal therapeutic use with physician approval (*n* = 1,113).

Do you support the possibility of patients growing medical cannabis for personal therapeutic use with physician approval?—“rather yes” or “definitely yes”				Bivariable logistic regression		Multivariable logistic regression	
Variable		%	*p*	OR (95%CI)	*p*	aOR (95%CI)	*p*
Gender	Female (*n* = 605)	40.2	**<0.001**	Reference	**<0.001**	Reference	**<0.001**
	Male (*n* = 508)	52.0		1.61 (1.27–2.05)		1.65 (1.29–2.11)	
Age group [years]	18–29 (*n* = 153)	53.6	**0.01**	1.84 (1.25–2.72)	**0.002**	1.75 (1.15–2.66)	**0.01**
	30–39 (*n* = 217)	50.7		1.64 (1.16–2.32)	**0.005**	1.46 (0.98–2.18)	0.06
	40–49 (*n* = 211)	46.0		1.36 (0.96–1.93)	0.09	1.21 (0.81–1.82)	0.3
	50–59 (*n* = 205)	44.9		1.30 (0.91–1.85)	0.1	1.25 (0.84–1.86)	0.3
	60 + (*n* = 327)	38.5		Reference		Reference	
Educational level	Higher (*n* = 536)	46.1	0.7	1.04 (0.82–1.32)	0.7		
	Less than higher (*n* = 577)	45.1		Reference			
Marital status	Single (*n* = 319)	48.3	0.1	Reference			
	Married (*n* = 580)	43.6		0.83 (0.63–1.09)	0.2		
	Informal relationship (*n* = 167)	50.3		1.08 (0.75–1.58)	0.7		
	Other (*n* = 47)	34.0		0.55 (0.29–1.05)	0.07		
Place of residence	Rural area (*n* = 419)	42.0	0.07	Reference		Reference	
	City below 20,000 residents (*n* = 145)	40.0		0.92 (0.63–1.35)	0.7	0.95 (0.64–1.41)	0.8
	City from 20,000 to 99,999 residents (*n* = 219)	48.9		1.32 (0.95–1.83)	0.1	1.33 (0.95–1.86)	0.1
	City from 100,000 to 499,999 residents (*n* = 187)	48.1		1.28 (0.91–1.81)	0.2	1.25 (0.88–1.79)	0.2
	City ≥ 500,000 residents (*n* = 143)	53.1		1.57 (1.07–2.29)	**0.02**	1.57 (1.06–2.32)	**0.02**
Number of household members	1 (living alone) (*n* = 191)	45.0	0.9	1.01 (0.72–1.41)	0.9		
	2 (*n* = 405)	46.7		1.08 (0.83–1.40)	0.6		
	3 or more (*n* = 517)	44.9		Reference			
Occupational status	Active (*n* = 695)	48.6	**0.01**	1.40 (1.09–1.78)	**0.01**	1.11 (0.82–1.50)	0.5
	Passive (*n* = 418)	40.4		Reference		Reference	
Self-reported household economic status	Good (*n* = 554)	46.5	0.2	0.94 (0.67–1.33)	0.7		
	Moderate (*n* = 396)	42.1		0.79 (0.61–1.02)	0.07		
	Bad (*n* = 142)	48.0		Reference			
Ever treatment with medical cannabis	Yes (*n* = 47)	72.3	**<0.001**	3.28 (1.71–6.28)	**<0.001**	2.93 (1.51–5.69)	**0.002**
	No (*n* = 1,066)	44.4		Reference		Reference	

OR – odds ratio; aOR – adjusted odds ratio. Bold values are statistically significant.

**Table 6:** Factors associated with public perception of physicians’ knowledge about medical cannabis therapy (*n* = 1,113).

In your opinion, do physicians in Poland have sufficient knowledge about medical cannabis therapy?—“rather yes” or “definitely yes”				Bivariable logistic regression		Multivariable logistic regression	
Variable		**%**	***p***	OR (95%CI)	*p*	aOR (95%CI)	*p*
Gender	Female (*n* = 605)	26.3	**0.005**	Reference	**0.005**	Reference	**0.004**
	Male (*n* = 508)	34.1		1.45 (1.12–1.88)		1.47 (1.13–1.91)	
Age group [years]	18–29 (*n* = 153)	31.4	0.8	1.07 (0.71–1.62)	0.8		
	30–39 (*n* = 217)	30.4		1.02 (0.70–1.48)	0.9		
	40–49 (*n* = 211)	31.3		1.06 (0.73–1.55)	0.7		
	50–59 (*n* = 205)	26.3		0.84 (0.57–1.24)	0.4		
	60 + (*n* = 327)	30.0		Reference			
Educational level	Higher (*n* = 536)	27.4	0.09	0.80 (0.62–1.04)	0.09		
	Less than higher (*n* = 577)	32.1		Reference			
Marital status	Single (*n* = 319)	27.9	0.8	1.14 (0.84–1.54)	0.4		
	Married (*n* = 580)	30.5		1.14 (0.75–1.71)	0.5		
	Informal relationship (*n* = 167)	30.5		1.21 (0.63–2.34)	0.6		
	Other (*n* = 47)	31.9		Reference			
Place of residence	Rural area (*n* = 419)	30.8	**0.03**	1.61 (1.03–2.52)	**0.04**	1.62 (1.03–2.55)	**0.04**
	City below 20,000 residents (*n* = 145)	26.2		1.28 (0.75–2.21)	0.4	1.32 (0.76–2.29)	0.3
	City from 20,000 to 99,999 residents (*n* = 219)	37.0		2.12 (1.31–3.44)	**0.002**	2.10 (1.28–3.43)	**0.003**
	City from 100,000 to 499,999 residents (*n* = 187)	28.3		1.43 (0.86–2.38)	0.2	1.36 (0.81–2.28)	0.2
	City ≥ 500,000 residents (*n* = 143)	21.7		Reference		Reference	
Number of household members	1 (living alone) (*n* = 191)	29.8	0.4	0.92 (0.64–1.31)	0.6		
	2 (*n* = 405)	27.4		0.81 (0.61–1.08)	0.2		
	3 or more (*n* = 517)	31.7		Reference			
Occupational status	active (*n* = 695)	31.2	0.2	1.20 (0.92–1.56)	0.2		
	Passive (*n* = 418)	27.5		Reference			
Self-reported household economic status	Good (*n* = 554)	29.7	0.7	0.94 (0.64–1.36)	0.7		
	Moderate (*n* = 396)	28.4		0.88 (0.66–1.17)	0.4		
	Bad (*n* = 142)	31.0		Reference			
Ever treatment with medical cannabis	Yes (*n* = 47)	66.0	**<0.001**	4.92 (2.66–9.13)	**<0.001**	4.94 (2.64–9.25)	**<0.001**
	No (*n* = 1,066)	28.2		Reference		Reference	

OR – odds ratio; aOR – adjusted odds ratio. Bold values are statistically significant.

**Table 7:** Factors associated with public perception of patients’ knowledge about medical cannabis therapy (*n* = 1,113).

In your opinion, do patients in Poland have sufficient knowledge about the potential use of medical cannabis therapy?—“rather yes” or “definitely yes”				Bivariable logistic regression		Multivariable logistic regression	
Variable		%	p	OR (95%CI)	p	aOR (95%CI)	p
Gender	Female (*n* = 605)	14.7	0.2	0.80 (0.58–1.10)	0.2		
	Male (*n* = 508)	17.7		Reference			
Age group [years]	18–29 (*n* = 153)	22.2	**0.004**	2.17 (1.31–3.62)	**0.003**	1.31 (0.73–2.34)	0.4
	30–39 (*n* = 217)	19.4		1.83 (1.13–2.94)	**0.01**	1.06 (0.60–1.87)	0.9
	40–49 (*n* = 211)	19.4		1.83 (1.14–2.97)	**0.01**	1.05 (0.58–1.88)	0.9
	50–59 (*n* = 205)	11.7		1.01 (0.59–1.74)	0.9	0.71 (0.39–1.31)	0.3
	60 + (*n* = 327)	11.6		Reference		Reference	
Educational level	Higher (*n* = 536)	14.6	0.2	0.80 (0.58–1.11)	0.2		
	Less than higher (*n* = 577)	17.5		Reference			
Marital status	Single (*n* = 319)	16.3	0.9	Reference			
	Married (*n* = 580)	16.0		0.98 (0.68–1.42)	0.9		
	Informal relationship (*n* = 167)	15.0		0.90 (0.54–1.52)	0.7		
	Other (*n* = 47)	19.1		1.22 (0.56–2.67)	0.6		
Place of residence	Rural area (*n* = 419)	16.0	**0.03**	1.41 (0.80–2.49)	0.2	1.35 (0.74–2.46)	0.3
	City below 20,000 residents (*n* = 145)	9.7		0.79 (0.38–1.67)	0.5	0.81 (0.37–1.77)	0.6
	City from 20,000 to 99,999 residents (*n* = 219)	19.6		1.81 (0.99–3.32)	0.06	1.72 (0.92–3.24)	0.09
	City from 100,000 to 499,999 residents (*n* = 187)	20.3		1.89 (1.02–3.51)	**0.04**	1.83 (0.96–3.48)	0.07
	City ≥ 500,000 residents (*n* = 143)	11.9		Reference		Reference	
Number of household members	1 (living alone) (*n* = 191)	15.2	**<0.001**	0.69 (0.44–1.09)	0.1	0.81 (0.49–1.32)	0.4
	2 (*n* = 405)	10.9		0.47 (0.32–0.69)	**<0.001**	0.56 (0.37–0.86)	**0.01**
	3 or more (*n* = 517)	20.5		Reference		Reference	
Occupational status	Active (*n* = 695)	18.7	**0.002**	1.73 (1.22–2.47)	**0.002**	1.48 (0.97–2.26)	0.07
	Passive (*n* = 418)	11.7		Reference		Reference	
Self-reported household economic status	Good (*n* = 554)	17.4	0.9	1.13 (0.71–1.78)	0.6		
	Moderate (*n* = 396)	15.9		1.00 (0.71–1.43)	0.9		
	Bad (*n* = 142)	15.8		Reference			
Ever treatment with medical cannabis	Yes (*n* = 47)	55.3	**<0.001**	7.39 (4.06–13.46)	**<0.001**	5.85 (3.15–10.86)	**<0.001**
	No (*n* = 1,066)	14.4		Reference		Reference	

OR – odds ratio; aOR – adjusted odds ratio. Bold values are statistically significant.

Age 50 and over (*p* < 0.05) was the only factor associated with declaration of positive opinion on the legalization of medical cannabis treatment for patients in Poland since 2017 (Table 3). Moderate household economic status was associated with low levels of satisfaction with the legalization of medical cannabis in 2017 (aOR: 0.51; 95%CI: 0.37–0.71; *p* < 0.001). Age 30–39 years (aOR: 0.33; 95%CI: 0.20–0.53; *p* < 0.001) and moderate household economic status (aOR: 0.51; 95%CI: 0.35–0.73; *p* < 0.001) were significantly associated with lower willingness to undergo medical cannabis therapy if recommended by the physician due to health conditions (Table 4). Male gender (aOR: 1.65; 95%CI: 1.29–2.11; *p* < 0.001), age 18–29 years (aOR: 1.15–2.26; *p* = 0.01), living in cities over 500,000 residents (aOR: 1.57; 95%CI: 1.06–2.32; *p* = 0.02), and reported prior treatment with medical cannabis (aOR: 2.93; 95%CI: 1.51–5.69; *p* = 0.002) were significantly associated with the support for the possibility of patients growing medical cannabis for personal therapeutic use with physician approval (Table 5).

Male gender (aOR: 1.47; 95%CI: 1.13–1.91; *p* = 0.004), living in rural area (aOR: 1.62; 95%CI: 1.03–2.55; *p* = 0.04) or cities from 20,000 to 99,999 residents (aOR: 2.10; 95%CI: 1.28–3.43; *p* = 0.003), and reported prior treatment with medical cannabis (aOR: 4.94; 95%CI: 2.64–9.25; *p* < 0.001) were significantly associated with opinion that physicians in Poland have sufficient knowledge about medical cannabis therapy (Table 6). A lifetime experience with medical cannabis treatment (aOR: 5.85; 95%CI: 3.15–10.86; *p* < 0.001) was the only factor significantly associated with the opinion that patients in Poland have sufficient knowledge about medical cannabis therapy (Table 7).

## 4 Discussion

This study presents a comprehensive analysis of public attitudes toward medical cannabis in Poland. The findings demonstrate that a substantial majority of respondents support the legalization of medical cannabis and indicate a willingness to consider such treatment if medically justified. Nevertheless, this expressed acceptance may not fully correspond to an informed or practical readiness to engage with cannabinoid-based therapies. A critical review by Silczuk et al. (15) highlights the terminological confusion surrounding medical and non-medical cannabis. Moreover, actual clinical exposure remains minimal, as reflected by the very low proportion of respondents reporting a physician’s recommendation for medical cannabis. This discrepancy raises important questions regarding whether public endorsement is grounded in accurate knowledge and personal experience, or rather influenced by a broader approval of policy liberalization. Additionally, these findings highlight the limited integration of medical cannabis into routine clinical practice in Poland, suggesting that favorable public opinion alone is insufficient to ensure effective implementation without concomitant systemic, clinical, and educational initiatives.

Real-world experiences during the early phase of the COVID-19 pandemic in the United States revealed that access to medical cannabis can be disrupted by external crises. While some patients reported stable or increased use, others experienced treatment interruptions due to reduced availability and limited healthcare support, emphasizing the need for resilient systems and adaptive regulatory frameworks (16). Similarly, even in routine settings, physician authorization does not guarantee use. A recent study from an academic medical center found that fewer than half of certified patients actually purchased cannabis, citing high costs, registration burdens, and limited access to dispensaries. Many therefore continued to rely on unregulated products, despite reporting symptom relief, as these options were more accessible and affordable (17).

Patient surveys conducted in dispensary settings have shown that individuals using medical cannabis for anxiety frequently report symptom improvement, minimal adverse effects, and high satisfaction with treatment. However, these findings should be interpreted with caution, as they are based on a self-selected population of active users and do not capture individuals who discontinued treatment. In addition, the surveys often lacked systematic data on product composition (e.g., THC versus CBD content) and did not clarify whether the reported benefits were sustained in the long term. Inhalation methods, especially vaporization, were most commonly used, and most patients perceived access as relatively easy and affordable (18). These self-reported findings suggest both high perceived efficacy and a preference for specific administration routes.

In contrast, clinician attitudes remain cautious. A systematic review revealed that although a majority of physicians report receiving patient requests for medical cannabis, a much smaller proportion are willing to prescribe it, largely due to insufficient clinical knowledge and concerns about efficacy, safety, and counseling (19). Importantly, this reluctance also reflects the broader absence of high-quality evidence, which stems from regulatory and medico-legal constraints, the mismatch with traditional pharmaceutical models, commercial barriers, and the heterogeneity of cannabis-based products studied across multiple conditions (15). Studies from Ontario similarly show that physicians reluctant to authorize medical cannabis often cite neurocognitive risks, psychiatric comorbidities, and lack of clear indications as primary reasons (20). Those with prior prescribing experience tend to be more confident in its therapeutic value, underscoring the importance of structured education and clinical exposure. At the national level, the Canadian Medical Association (CMA) has also highlighted such concerns in its policy statement on cannabis for medical purposes, particularly emphasizing the limited scientific evidence, medico-legal liability, and challenges tied to pharmaceutical regulation and prescribing frameworks (21).

Our findings reflect this disconnect. Although public support is high, fewer than one third of respondents believe that physicians in Poland possess sufficient knowledge about medical cannabis, and even fewer trust that patients are adequately informed. These perceptions are consistent with Canadian research, where users report therapeutic benefits and reduced use of opioids and sedatives, yet face barriers such as limited provider guidance and reliance on alternative information sources (17, 22). A study from Thailand further confirmed that healthcare workers and health volunteers differ in training needs and attitudes, pointing to the necessity of role-specific education tailored to local contexts (23). Comparable challenges have been documented in other jurisdictions: recent Israeli, Canadian and North American data underscore the growing demand for evidence-based physician training and system-level guidance (24–30), while Australian and New Zeelands studies highlight both therapeutic potential and persistent regulatory and clinical barriers to implementation (31, 32). Together, these findings suggest that despite cultural and policy differences, the need for structured education and clear regulatory frameworks is a common international theme. This becomes particularly evident when public perceptions are contrasted with legislatively defined frameworks. In Poland, for example, the law does not specify a closed list of indications, yet respondents most frequently mentioned cancer and chronic pain conditions that also rank among the most common reasons for medical cannabis authorization in other countries.

Surveys from different regions likewise reveal large gaps in clinical preparedness. In Florida, certifiers reported reliance on dispensary staff and inconsistent communication with other providers, as well as a strong need for structured education on interactions, condition-specific evidence, and harm reduction strategies (33). A cross-sectional study of medical cannabis providers revealed similar variability in practice, including inconsistent dosing guidance and reliance on self-directed learning in the absence of formal guidelines. Most patients were cannabis-naïve, placing a significant educational burden on clinicians (34). Polish data mirror these concerns. Many physicians lack knowledge of the legal framework, pharmacology, and clinical effects of cannabinoids. Male physicians and those with less professional experience tended to score higher in knowledge assessments, but overall familiarity was low (35).

International experience illustrates the impact of regulatory design. In Canada, requiring physician authorization for access to licensed producers initially led to higher patient enrollment in the medical program, as this was the only legal route to obtain cannabis before the legalization of non-medical use in 2018. Following legalization, cannabis became widely accessible to all adults, which reduced the gatekeeping role of physicians and was accompanied by a decline in medical program participation. Nonetheless, Canadian data suggest that formal medical authorization still contributes to safer, better-informed use: authorized users were more likely to obtain regulated products, report fewer side effects, and consult healthcare professionals (36, 37).

Attitudes toward medical cannabis were influenced by sociodemographic factors. In our study, respondents over 50 were more likely to support legalization. Interestingly, those aged 30 to 39 expressed lower willingness to accept medical cannabis treatment. Individuals with moderate economic status were less supportive than those reporting low income, which may reflect differences in access to information or health services. Participants were most familiar with the use of medical cannabis in oncological, neurological, and chronic pain conditions, while psychiatric and inflammatory indications were less recognized. This suggests a need for public education on the full therapeutic scope. Support for home cultivation was associated with prior experience, male gender, younger age, and urban residence. These findings point to experiential and demographic factors shaping views on patient autonomy in cannabis use. Patient perspectives reinforce the importance of perceived efficacy and symptom relief (38). In Florida, most patients reported high satisfaction with medical cannabis use for conditions like chronic pain, depression, and arthritis, though cost remained a common concern (39). A California study tracking transitions in medical cannabis patient status found that individuals who maintained status used more cannabis but reported lower use of other drugs, whereas those who discontinued primarily reported reduced cannabis use. While these findings have been interpreted as suggesting a potential harm reduction role for medical cannabis, the evidence remains limited and should be viewed with caution given the observational design and reliance on self-reported data (40).

Beliefs about system-level competence were associated with prior experience, male gender, and non-urban residence. These patterns may reflect either greater exposure to cannabis or perceptual bias among users. A study of New Zealand physicians found that prescribing decisions were influenced by product quality, side-effect profiles, patient–provider relationships, and concerns about professional liability and cost barriers. Clinicians emphasized the need for clear guidelines to support ethical, equitable prescribing (41).

Finally, the 2023 Cannabis Clinical Outcomes Research Conference (CCORC) highlighted major themes in medical cannabis research, including methodological challenges, the need to clarify risks and benefits, and data gaps in vulnerable populations. Current shortcomings in the evidence base include the limited number of high-quality randomized controlled trials, small and heterogeneous study populations, and insufficient data on long-term safety and effectiveness across diverse patient groups. To address these gaps, both pragmatic real-world evidence studies and traditional clinical trials are needed, with complementary strengths in external validity and methodological rigor. Ongoing research and robust clinical data will be essential to guide evidence-based policymaking and medical education (42).

## 5 Conclusion

The study demonstrates that public support for medical cannabis in Poland is high. Most respondents expressed favorable attitudes toward its legalization and indicated willingness to undergo such therapy if medically indicated. At the same time, there is a pronounced lack of confidence in the knowledge of both physicians and patients regarding medical cannabis treatment.

Sociodemographic factors such as age, gender, economic status, urbanicity, and prior experience with medical cannabis were significantly associated with public attitudes. Older individuals and those with previous therapeutic use were more supportive, while individuals with moderate economic status showed lower acceptance.

These findings suggest a disconnect between the level of public endorsement and perceived readiness of the healthcare system to deliver cannabis-based treatments. Addressing informational deficits among both healthcare professionals and the general population may be critical to ensure safe and effective implementation of medical cannabis policies in Poland. In parallel, high-quality clinical trials and pragmatic real-world evidence studies are urgently needed to generate the robust data required for the development of evidence-based guidelines and educational initiatives for both physicians and patients.

## 6 Limitations

This cross-sectional survey has several methodological limitations that should be considered when interpreting the findings. First, all data were self-reported by respondents and may be subject to reporting biases, including social desirability and recall bias. Participants’ attitudes, beliefs, and declared knowledge about medical cannabis were not independently verified against clinical data or objective measures of health literacy. Second, the study does not assess the real-world effectiveness of cannabis policy implementation or its clinical outcomes. Given its cross-sectional design and absence of longitudinal or experimental comparison, the study cannot establish causal relationships between public attitudes and actual patterns of access, prescription, or use. Third, while the CAWI technique enabled efficient and demographically representative data collection across Poland, it inherently excluded individuals without internet access or those unfamiliar with online survey tools, potentially limiting generalizability to digitally excluded populations. Finally, physicians and nurses were not surveyed, meaning that the findings reflect only public perceptions of healthcare professionals’ knowledge and experience with medical cannabis, rather than the perspectives of clinicians themselves.

Nonetheless, this study offers a timely and policy-relevant snapshot of public perceptions surrounding medical cannabis in Poland and may serve as a foundation for future longitudinal or intervention-based research. Especially on cannabis policy implementation or its clinical outcomes.

## Data Availability

The raw data supporting the conclusions of this article will be made available by the authors, without undue reservation.

## References

[1] Wang RQ Bonomo YA Hallinan CM. Overview of global monitoring systems for the side effects and adverse events associated with medicinal cannabis use: a scoping review using a systematic approach. BMJ Open. (2024) 14:e085166. doi: 10.1136/bmjopen-2024-085166, PMID: 39025811 PMC11733909

[2] Hordowicz M Jarosz J Czaplińska M Leonhard A Klimkiewicz A. Polish physicians' perspectives on medical Cannabis policy and educational needs: results of an online survey. J Clin Med. (2021) 10:4545. doi: 10.3390/jcm10194545, PMID: 34640561 PMC8509273

[3] Wang Q Qin Z Xing X Zhu H Jia Z. Prevalence of cannabis use around the world: a systematic review and me-ta-analysis, 2000-2024. China CDC Wkly. (2024) 6:597–604. doi: 10.46234/ccdcw2024.116 38933041 PMC11196877

[4] Los G. Taking stock - legalization of polish medicinal cannabis seven years later: users' perspectives. Subst Use Misuse. (2025) 60:522–30. doi: 10.1080/10826084.2024.2434683, PMID: 39612202

[5] Hordowicz MJ Jarosz J Klimkiewicz A Czaplińska M Leonhard A Wysocka M. To treat or not to treat? Polish Phy-sicians' opinions about the clinical aspects of cannabinoids-an online survey. J Clin Med. (2022) 11:236. doi: 10.3390/jcm11010236, PMID: 35011977 PMC8745737

[6] Śmiarowska M Białecka M Machoy-Mokrzyńska A. Cannabis and cannabinoids: pharmacology and ther-apeutic potential. Neurol Neurochir Pol. (2022) 56:4–13. doi: 10.5603/PJNNS.a2022.0015, PMID: 35133644

[7] Gujska JH Silczuk A Madejek R Szulc A. Exploring the link between attention-deficit hyperactivity disorder and Cannabis use disorders: a review. Med Sci Monit. (2023) 29:e939749. doi: 10.12659/MSM.939749, PMID: 37147797 PMC10171029

[8] Schlag AK. An evaluation of regulatory regimes of medical Cannabis: what lessons can be learned for the UK? Med Cannabis Cannabinoids. (2020) 3:76–83. doi: 10.1159/000505028, PMID: 34676342 PMC8489334

[9] Bramness JG Dom G Gual A Mann K Wurst FM. A survey on the medical use of Cannabis in Europe: a position paper. Eur Addict Res. (2018) 24:201–5. doi: 10.1159/000492757, PMID: 30134238

[10] Russo E Cannas C Rivetti MS Villa C Rebesco B. Innovative clinical-organizational model to ensure appropriateness and quality in the Management of Medical Cannabis: an Italian regional case. Healthcare (Basel). (2021) 9:1425. doi: 10.3390/healthcare9111425, PMID: 34828472 PMC8625658

[11] Farber Y Nir O Farber S. Medicalization without legalization: the European policy for medical and recreational cannabis use. J Public Health. (2024). doi: 10.1007/s10389-024-02368-y

[12] Mularczyk-Tomczewska P Lewandowska A Kamińska A Gałecka M Atroszko PA Baran T. Patterns of energy drink use, risk perception, and regulatory attitudes in the adult polish population: results of a cross-sectional survey. Nutrients. (2025) 17:1458. doi: 10.3390/nu17091458, PMID: 40362767 PMC12073747

[13] Lewandowska A Jankowski M Gujski M Mularczyk-Tomczewska P Szulc A Silczuk A. Self-reported help-seeking behavior in the event of mental disorders among adults in Poland. Front Public Health. (2025) 13:1612066. doi: 10.3389/fpubh.2025.1612066, PMID: 40575095 PMC12197956

[14] Silczuk A Lewandowska A Baran T Koweszko T Mularczyk-Tomczewska P Gujska J. Sociocultural correlates and epidemiological patterns of non-alcoholic beer consumption: a cross-sectional study in Poland. BMJ Open. (2025) 15:e100408. doi: 10.1136/bmjopen-2025-100408, PMID: 40730394 PMC12306457

[15] Silczuk A Smułek D Kołodziej M Gujska J. The construct of medical and non-medical marijuana-critical review. Int J Environ Res Public Health. (2022) 19:2769. doi: 10.3390/ijerph19052769, PMID: 35270462 PMC8910105

[16] Lake S Assaf RD Gorbach PM Cooper ZD. Selective changes in medical Cannabis use early in the COVID-19 pandemic: findings from a web-based sample of adults in the United States. Cannabis Cannabinoid Res. (2023) 8:174–83. doi: 10.1089/can.2021.0115, PMID: 35073161 PMC9940798

[17] Cahill SP Lunn SE Diaz P Page JE. Evaluation of patient reported safety and efficacy of Cannabis from a survey of medical Cannabis patients in Canada. Front Public Health. (2021) 9:626853. doi: 10.3389/fpubh.2021.626853, PMID: 34095048 PMC8172603

[18] Kimless D Caloura M Markos V Ryan J Abbonizio S Janicki S. An observational cross-sectional survey exploring the indications for and responses to medical marijuana use in certified patients in Pennsylvania. J Prim Care Community Health. (2022) 13:21501319221129734. doi: 10.1177/21501319221129734, PMID: 36226444 PMC9561639

[19] Rønne ST Rosenbæk F Pedersen LB Waldorff FB Nielsen JB Riisgaard H. Physicians' experiences, at-titudes, and beliefs towards medical cannabis: a systematic literature review. BMC Fam Pract. (2021) 22:212. doi: 10.1186/s12875-021-01559-w, PMID: 34674661 PMC8532330

[20] Ng JY Gilotra K Usman S Chang Y Busse JW. Attitudes toward medical cannabis among family physicians practising in Ontario, Canada: a qualitative research study. CMAJ Open. (2021) 9:E342–8. doi: 10.9778/cmajo.20200187, PMID: 33849983 PMC8084545

[21] Canadian Medical Association. Medical Marijuana Policy Statement [Internet]. Ottawa: Canadian Medical Association (2003).

[22] Butler JI Dahlke S Devkota R Shrestha S Hunter KF Toubiana M. The Infor-mation-seeking behavior and unmet knowledge needs of older medicinal Cannabis consumers in Canada: a Quali-tative descriptive study. Drugs Aging. (2023) 40:427–38. doi: 10.1007/s40266-023-01030-8, PMID: 37147415 PMC10162651

[23] Mekrungrongwong S Kitreerawutiwong N Keeratisiroj O Jariya W. Self-perceived knowledge, attitudes, and training needs regarding medical cannabis among health care providers and health volunteers in district health systems, Phitsanulok Province. BMC Prim Care. (2022) 23:266. doi: 10.1186/s12875-022-01877-7, PMID: 36271334 PMC9585781

[24] Zolotov Y Edelstein OE Temple LM Kogan M Romem-Porat SL Reznik A. Education, training, and perceptions of physician competency among medical cannabis patients in Israel. Complement Ther Med. (2025) 90:103172. doi: 10.1016/j.ctim.2025.103172, PMID: 40185288

[25] HaGani N Sznitman S Dor M Bar-Sela G Oren D Margolis-Dorfman L. Attitudes toward the use of medical cannabis and the perceived efficacy, side-effects and risks: a survey of patients, nurses and physicians. J Psychoactive Drugs. (2022) 54:393–402. doi: 10.1080/02791072.2021.2009598, PMID: 34893011

[26] St Pierre M Matthews L Walsh Z. Cannabis education needs assessment among Canadian physicians-in-training. Complement Ther Med. (2020) 49:102328. doi: 10.1016/j.ctim.2020.102328, PMID: 32147035

[27] Kruger DJ Gerlach J Kruger JS Mokbel MA Clauw DJ Boehnke KF. Physicians' attitudes and practices regarding Cannabis and recommending medical Cannabis use. Cannabis Cannabinoid Res. (2024) 9:e1048–55. doi: 10.1089/can.2022.0324, PMID: 37098170 PMC11538087

[28] Worster B Ashare RL Hajjar E Garber G Smith K Kelly EL. Clinician attitudes, training, and beliefs about Cannabis: an Interprofessional assessment. Cannabis Cannabinoid Res. (2023) 8:547–56. doi: 10.1089/can.2021.0022, PMID: 34978882

[29] Takakuwa KM Mistretta A Pazdernik VK Sulak D. Education, knowledge, and practice characteristics of Cannabis physicians: a survey of the Society of Cannabis Clinicians. Cannabis Cannabinoid Res. (2021) 6:58–65. doi: 10.1089/can.2019.0025, PMID: 33614953 PMC7891199

[30] Syed SA Singh J Elkholy H Palavra IR Tomicevic M Eric AP. International perspective on physician knowledge, attitude and practices related to medical cannabis. medRxiv [Preprint] (2023). doi: 10.1101/2023.07.26.23293157 PMC11882600 40051509

[31] Graham M Renaud E Lucas CJ Schneider J Martin JH. Medicinal Cannabis guidance and resources for health professionals to inform clinical decision making. Clin Ther. (2023) 45:527–34. doi: 10.1016/j.clinthera.2023.03.007, PMID: 37414503

[32] Withanarachchie V Rychert M Wilkins C. Barriers and facilitators to prescribing medicinal cannabis in New Zealand. J Prim Health Care. (2023) 15:135–46. doi: 10.1071/HC22122, PMID: 37390030

[33] Sajdeya R Shavers A Jean-Jacques J Costales B Jugl S Crump C. Practice patterns and training needs among physicians certifying patients for medical marijuana in Florida. J Prim Care Community Health. (2021) 12:21501327211042790. doi: 10.1177/21501327211042790, PMID: 34452585 PMC8404623

[34] Corroon J Sexton M Bradley R. Indications and administration practices amongst medical cannabis healthcare providers: a cross-sectional survey. BMC Fam Pract. (2019) 20:174. doi: 10.1186/s12875-019-1059-8, PMID: 31837706 PMC6911295

[35] Florek Łuszczki M Choina P Lachowski S Chmielewski J Łuszczki JJ. Medical marijuana – knowledge and opinions of primary care physicians in Lublin province, Poland. Ann Agric Environ Med. (2025) 32:255–61. doi: 10.26444/aaem/204099 40586503

[36] Shim M Nguyen H Grootendorst P. Lessons from 20 years of medical cannabis use in Canada. PLoS One. (2023) 18:e0271079. doi: 10.1371/journal.pone.0271079, PMID: 36952564 PMC10035846

[37] Balneaves LG Brown A Green M Prosk E Rapin L Monahan-Ellison M. Canadians' use of cannabis for therapeutic purposes since legalization of recreational cannabis: a cross-sectional analysis by medical authorization status. BMC Med. (2024) 22:150. doi: 10.1186/s12916-024-03370-7, PMID: 38589855 PMC11003000

[38] Jankie S Sewdass K Smith W Naraynsingh C Johnson J Farnon N. A cross-sectional survey of prospective healthcare professionals' knowledge, attitudes and perceptions of medical Cannabis. Explor Res Clin Soc Pharm. (2023) 10:100275. doi: 10.1016/j.rcsop.2023.100275, PMID: 37168830 PMC10165452

[39] Luque JS Okere AN Reyes-Ortiz CA Williams PM. Mixed methods study of the potential therapeutic benefits from medical cannabis for patients in Florida. Complement Ther Med. (2021) 57:102669. doi: 10.1016/j.ctim.2021.102669, PMID: 33460744 PMC8053674

[40] Fedorova EV Ataiants J Wong CF Iverson E Lankenau SE. Changes in medical cannabis patient status before and after cannabis legalization in California: associations with cannabis and other drug use. J Psychoactive Drugs. (2022) 54:129–39. doi: 10.1080/02791072.2021.1926604, PMID: 34044753 PMC8626530

[41] Manoharan R Kemper J Young J. Exploring the medical cannabis prescribing behaviours of New Zealand physicians. Drug Alcohol Rev. (2022) 41:1355–66. doi: 10.1111/dar.13476, PMID: 35604868 PMC9544511

[42] Goodin AJ Tran PT McKee S Sajdeya R Jyot J Cook RL. Proceedings of the 2023 Cannabis clinical outcomes research conference. Med Cannabis Cannabinoids. (2023) 6:97–101. doi: 10.1159/000533943., PMID: 37900895 PMC10601943

